# Iodine Promoted Ultralow Zn Nucleation Overpotential and Zn-Rich Cathode for Low-Cost, Fast-Production and High-Energy Density Anode-Free Zn-Iodine Batteries

**DOI:** 10.1007/s40820-022-00948-9

**Published:** 2022-10-26

**Authors:** Yixiang Zhang, Lequan Wang, Qingyun Li, Bo Hu, Junming Kang, Yuhuan Meng, Zedong Zhao, Hongbin Lu

**Affiliations:** 1 State Key Laboratory of Molecular Engineering of Polymers, Department of Macromolecular Science, Fudan University, 2005 Songhu Road, Shanghai, 200438 People’s Republic of China; 2 Yiwu Research Institute of Fudan University, Chengbei Road, Yiwu City, Zhejiang, 322000 People’s Republic of China

**Keywords:** Zn metal battery, Zn deposition, Zn-rich cathode, Anode-free, Energy density

## Abstract

The anode-free design is a promising strategy to increase the energy density of aqueous Zn metal batteries (AZMBs). However, the scarcity of Zn-rich cathodes and the rapid loss of limited Zn greatly hinder their commercial applications. To address these issues, a novel anode-free Zn-iodine battery (AFZIB) was designed via a simple, low-cost and scalable approach. Iodine plays bifunctional roles in improving the AFZIB overall performance: enabling high-performance Zn-rich cathode and modulating Zn deposition behavior. On the cathode side, the ZnI_2_ serves as Zn-rich cathode material. The graphene/polyvinyl pyrrolidone heterostructure was employed as an efficient host for ZnI_2_ to enhance electron conductivity and suppress the shuttle effect of iodine species. On the anode side, trace I_3_^−^ additive in the electrolyte creates surface reconstruction on the commercial Cu foil. The in situ formed zincophilic Cu nanocluster allows ultralow-overpotential and uniform Zn deposition and superior reversibility (average coulombic efficiency > 99.91% over 7,000 cycles). Based on such a configuration, AFZIB exhibits significantly increased energy density (162 Wh kg^−1^) and durable cycle stability (63.8% capacity retention after 200 cycles) under practical application conditions. Considering the low cost and simple preparation methods of the electrode materials, this work paves the way for the practical application of AZMBs.

**Supplementary Information:**

The online version contains supplementary material available at 10.1007/s40820-022-00948-9.

## Highlights

- The I_3_^−^ additive promoted in situ formation of zincophilic Cu nanoclusters on commercial Cu foil, which achieves uniform Zn deposition with ultralow nucleation overpotential and high reversibility.
- The Zn-rich ZnI_2_ cathode confined by graphene/polyvinyl pyrrolidone heterostructure shows enhanced conductivity and shuttle effect suppression, providing sufficient Zn^2+^ for the anode stably.
- The assembled anode-free Zn-iodine battery exhibits attractive features for commercialization: low cost, fast production, significantly increased energy density and durable cycle stability under practical application conditions.

**Supplementary Information:**

The online version contains supplementary material available at 10.1007/s40820-022-00948-9.

## Graphical abstract

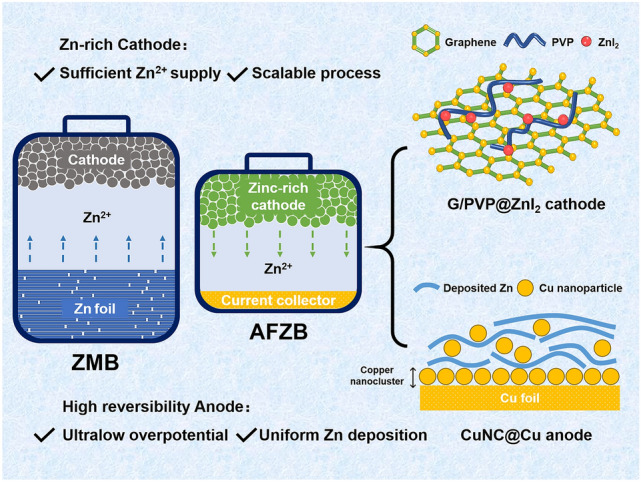

## Introduction

Subject to the safety and limited availability of lithium metal [1], researchers have increasingly focused on the development of new battery systems, such as sodium, potassium, zinc, and magnesium batteries. Among them, rechargeable Zn metal batteries (RZMBs) exhibit excellent safety with their unique aqueous electrolyte, while combining low cost and high specific capacity [2]. These advantages make RZMBs an important complement to lithium batteries, especially in the field of large-scale energy storage [3]. Although the performance of RZMBs has been greatly improved, the instability [4] of the Zn anodes is still the bottleneck restricting their commercialization [5, 6]. One of the important and unresolved issues is that previous reports generally use thick Zn foils (> 100 μm) as the anode [7], which leads to high negative to positive capacity ratios (N/P > 50) [8]. The large excess of Zn can compensate for the rapid loss of Zn during cycling to achieve excellent cell performance [9]. Nevertheless, these performances cannot be used as references for practical applications because the imbalanced N/P ratio will greatly limit the actual energy density of RZMBs [10]. Therefore, achieving RZMBs with low N/P ratio is critical to advancing practical progress.

As a hostless anode, the thinner Zn foil tends to fragment and pulverize during deep discharge process, resulting in severe surface corrosion and even forming isolated dead Zn [11]. Due to the limited amount of Zn, the thin Zn foil (10 μm) cannot release its theoretical capacity upon cycling, thus it is difficult to match the cathode capacity to assemble a low N/P ratio full cell. In current studies, low N/P ratios are controlled by electrochemically pre-depositing limited Zn on conductive substrates [12]. Such a costly and complex process is only suitable for laboratory-level studies. A more promising strategy is the anode-free Zn batteries (AFZBs) [13]. The Zn-rich cathode materials serve as the Zn source, and the Zn metal anode is replaced by a deposition substrate [14]. Due to the absence of Zn foils, this strategy maximizes the energy density of the battery.

However, achieving high-performance AFZBs is more challenging. On the anode side, because there is no excess Zn, corrosion, dendrites and side reactions [15] would lead to large amounts of Zn loss and accelerate capacity degradation. As a result, the AFZBs demonstrate a very short cycle life [10]. Therefore, suppressing Zn loss and improving the Coulombic efficiency (CE) of Zn deposition/dissolution are the keys to prolonging battery lifespan. In the current research works of AFZBs, Cui et al. introduced carbon disks on the copper (Cu) substrate as uniform nucleation sites for Zn, achieving a CE as high as 99.6% [16] in the 3 M Zn(CF_3_SO_3_)_2_ electrolyte. Feng et al. employed a silver-coated Cu substrate and 3 M Zn(TFSI)_2_ electrolyte to achieve a CE of 99.86% [17]. However, the expensive electrolytes and complex electrode processes used in these efforts greatly increase the cost of full cells, which reduces the commercialization potential of AFZBs. Therefore, it is necessary to further explore low-cost, scalable methods to improve Zn deposition/dissolution CE.

On the cathode side, unlike various types of Li-rich cathodes [18], such as lithium iron phosphate (LiFePO_4_), lithium manganate (LiMn_2_O_4_) and lithium cobaltate (LiCoO_2_), there are few reported high-performance Zn-rich cathodes [19]. For example, the Zn manganate (ZnMn_2_O_4_) cathode possesses a complete spinel structure, resulting in slow kinetics of Zn^2+^ extraction, poor reversibility and low specific capacity [20]; the ZnCo_2_O_4_ cathode is unstable in aqueous electrolytes and requires appropriate lattice modification (e.g., Al insertion [21]) to improve its stability. Furthermore, in some AFZB studies, Zn-rich manganese cathodes were obtained by pre-zincificated β-MnO_2_ [16], but such a complex and unstable procedure is unsuitable to scale up in practical applications. In this context, Zn-rich cathodes with stable Zn^2+^ supply and simple processes are urgently needed for the development of AFZBs.

**Fig. 1:**
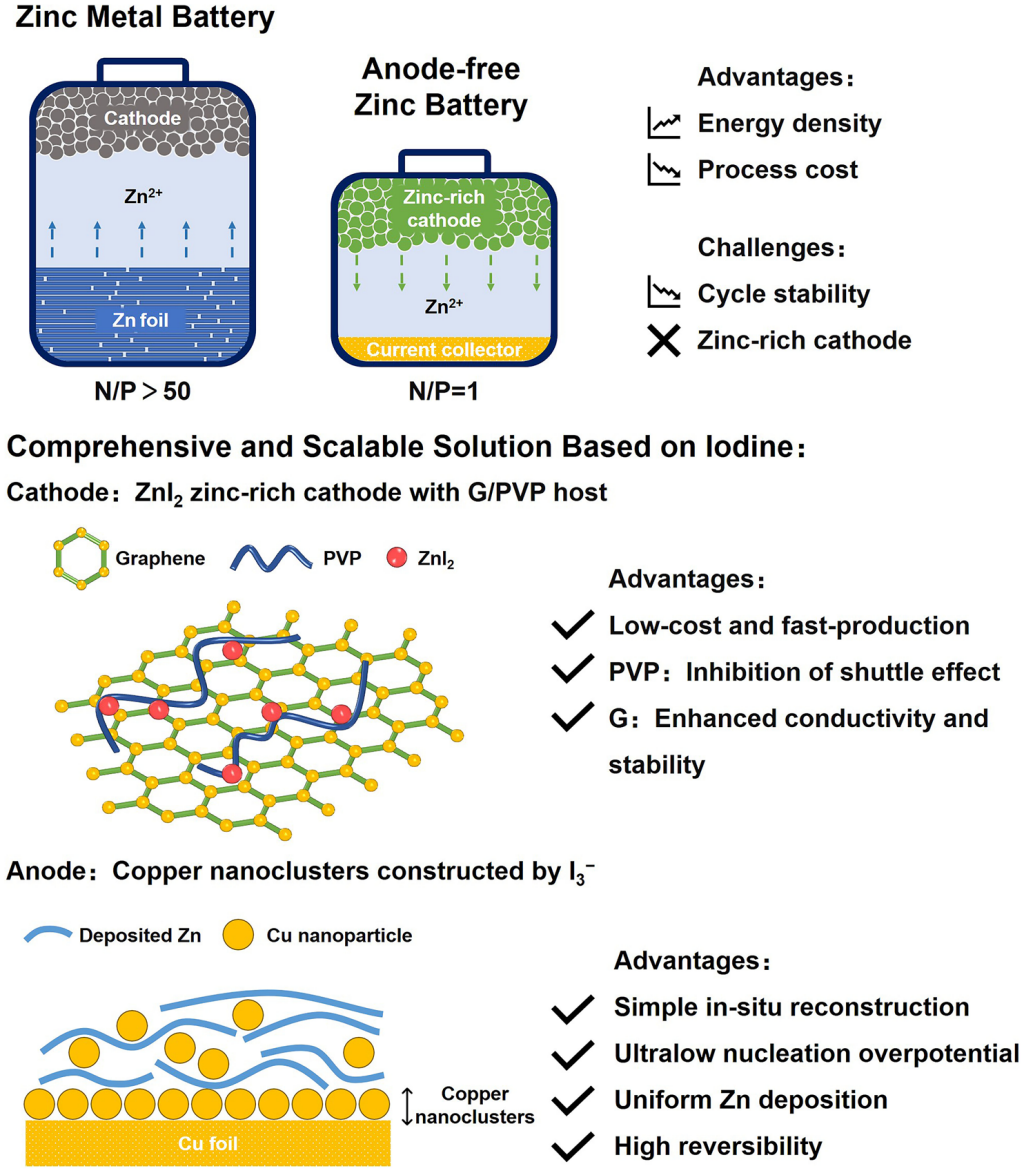
Advantages and challenges for anode-free Zn metal batteries and the comprehensive solution based on iodine proposed in this work

Herein, from the perspective of overcoming both the cathode and anode issues of AFZBs, we report a novel, low-cost, scale-up concept to design anode-free Zn-iodine battery (AFZIB) with high energy density and long lifespan (Fig. 1). On the cathode side, we propose a ZnI_2_ cathode and design a unique graphene/polyvinyl pyrrolidone (G/PVP) heterostructure as the host. PVP can effectively inhibit the shuttle effect of iodine species, while G can enhance the electronic conductivity and structure stability. Therefore, the G/PVP@ZnI_2_ cathode can deliver high specific capacity (146.1 mAh g^−1^_ZnI2_ at 1 A g^−1^, all the specific capacities are based on the mass of ZnI_2_) and excellent cycling performance (80% capacity retention after 1,000 cycles at 1 A g^−1^). On the anode side, we employed Cu foil as the deposition substrate and added a trace amount of I_3_^−^ (10 mM) to the electrolyte, which reacted with Cu surface to produce copper iodide (CuI). The CuI was reduced and reconstructed into Cu nanoclusters (CuNC) via in situ electrochemical reduction, which can modulate the Zn deposition behavior with ultralow nucleation overpotential and uniform morphology. The CE is ultra-high and stable with an average value > 99.91% for 7,000 cycles. Due to the multiple functions of iodine at both cathodes and anodes, the AFZIB exhibits excellent cycle stability (capacity retention rate of 63.8% after 200 cycles) and high specific capacity (125.7 mAh g^−1^ at 1 A g^−1^). More importantly, based on practical application level, the energy density of our AFZIB is significantly improved (162 Wh kg^−1^, based on active material), compared to traditional ZIBs (15 Wh kg^−1^ with a N/P of 50).

## Experimental Section

### Preparation of G/PVP@ZnI_2_ Cathode

The G was prepared according to our previously reported method [22]. For the G/PVP@ZnI_2_ cathode fabrication, the ZnI_2_, G, PVP (with an average molecular weight of 20,000) and polytetrafluoroethylene (PTFE) were mixed in ethanol at a mass ratio of 5:2:2:1 and ultrasonic dispersion for 30 min, followed by compressing onto a Ti grid. The electrodes were dried at 60 ℃ for 1 h and then punched into disks. The mass loadings of active materials were around 4–5 mg cm^−2^. The G@ZnI_2_ cathode was prepared by the same method with ZnI_2_, G and PTFE ratio of 5:4:1.

### Electrochemical Measurements

The I_3_^−^ containing electrolyte (10 mM) was prepared by adding 5 mM ZnI_2_ and 10 mM I_2_ into the 2 M ZnSO_4_ electrolyte. During the battery assembly process, the I_3_^−^ containing electrolyte first contacted and fully reacted with the commercial Cu foil before loading the Zn foil.

All the batteries were assembled with 2016-coin cells, and the separator was the glass fiber diaphragm (100 μm, Whatman GF/A) with an electrolyte addition of 70 µL (if not otherwise specified). Galvanostatic discharging/charging tests were performed on the Land battery tester (CT2001A) at various current densities. The cyclic voltammetry (CV) measurements were performed on a CHI660E electrochemical workstation.

### Material Characterizations

The structure and morphology of materials were characterized by field-emission scanning electron microscope (FESEM, Ultra 55). The crystal structure was measured with an X-ray diffractometer (XRD) model of PAN alytial X’Pert PRO. X-ray photoelectron spectroscopy (XPS) was measured using AXIS ULTRA DLD to obtain the surface chemical state of materials. The UV absorption spectra were measured on a Lambda750 UV–vis spectrophotometer.

## Results and Discussion

### In situ Surface Reconstruction of Cu Foil

**Fig. 2:**
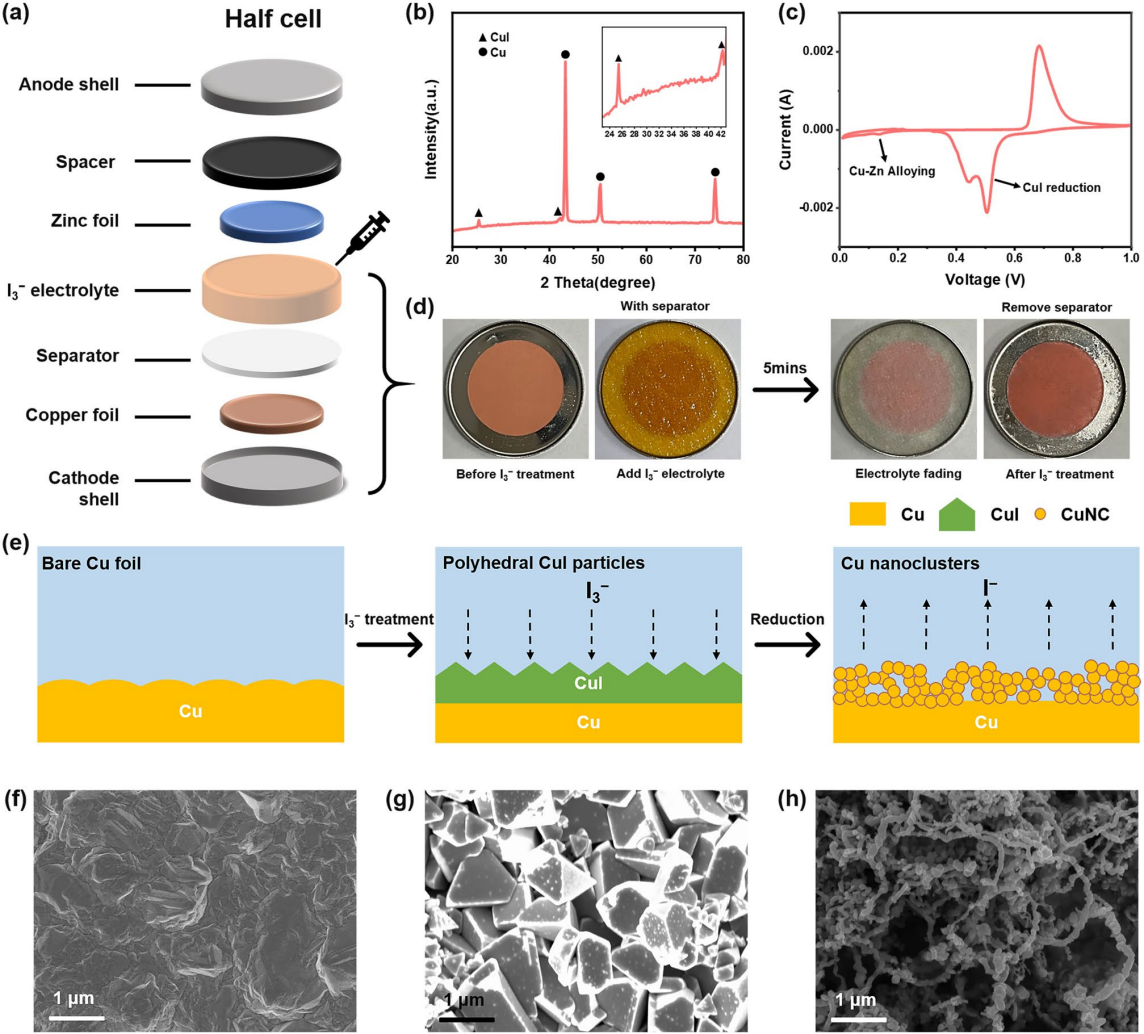
**a** Schematic illustration of the assembly process of half cell. **b** XRD pattern of the iodine-treated Cu foil. **c** CV curves of the CuI-Zn half cell at 0.2 mV s^−1^ with the voltage range of 0.01–1 V. **d** Digital photographs of the reaction process between Cu foil and I_3_^−^ electrolyte during the cell assembly process. **e** Schematic diagram of the surface morphological changes of the Cu foil during in situ reconstitution and the corresponding SEM images of Cu foil **f** before I_3_^−^ treatment, **g** after I_3_^−^ treatment and **h** after reduction to 0.1 V

As a low-cost and stable current collector for commercial lithium-ion batteries, Cu foil is also considered an ideal deposition substrate for Zn anodes in ZMBs [23]. For example, the recently reported Cu nanowires [24] or carbon fibers doped with Cu nanoboxes [12] show highly zincophilic features, which achieves uniform Zn deposition [25] with high CE. However, the finely designed Cu nanostructures in these efforts often require high costs and complex synthesis processes, such as the hydrothermal method [24] or high-temperature calcination [12]. Inspired by the previously reported study that employed I_3_^−^ to suppress hydrogen evolution of Zn anodes [26], we thus propose to use I_3_^−^ electrolyte additive to obtain zincophilic Cu nanocluster structures on the surface of commercial Cu foils via a simple in situ electrochemical reduction approach. The surface reconstruction process is simple and fast. Firstly, a trace amount of I_3_^−^ ions (10 mM) was added to the 2 M ZnSO_4_ aqueous electrolyte. Due to the strong oxidizing properties of I_3_^−^, the Cu foil reacted with the I_3_^−^ rapidly to produce CuI on its surface. Figure 2a, d shows the in situ reaction process between I_3_^−^ and Cu foil during the cell assembly process. After adding electrolyte to the separator (the separator ensures the uniform reaction between I_3_^−^ and Cu foil surface), the rufous electrolyte faded within 5 min, corresponding to the color change from pink to purplish red on the surface of the Cu foil. In the XRD pattern of the iodine-treated Cu foil (Fig. 2b), the characteristic peaks at 25.4° and 42.3° are attributed to the (111) and (220) crystal planes of CuI [27], respectively. The high-resolution XPS spectra verified the strong Cu^1+^2*p*_3/2_ (932.3 eV) and Cu^1+^2*p*_1/2_ (932.3 eV) peaks [28] (Fig. S1), which also indicate the existence of CuI.

**Fig. 3:**
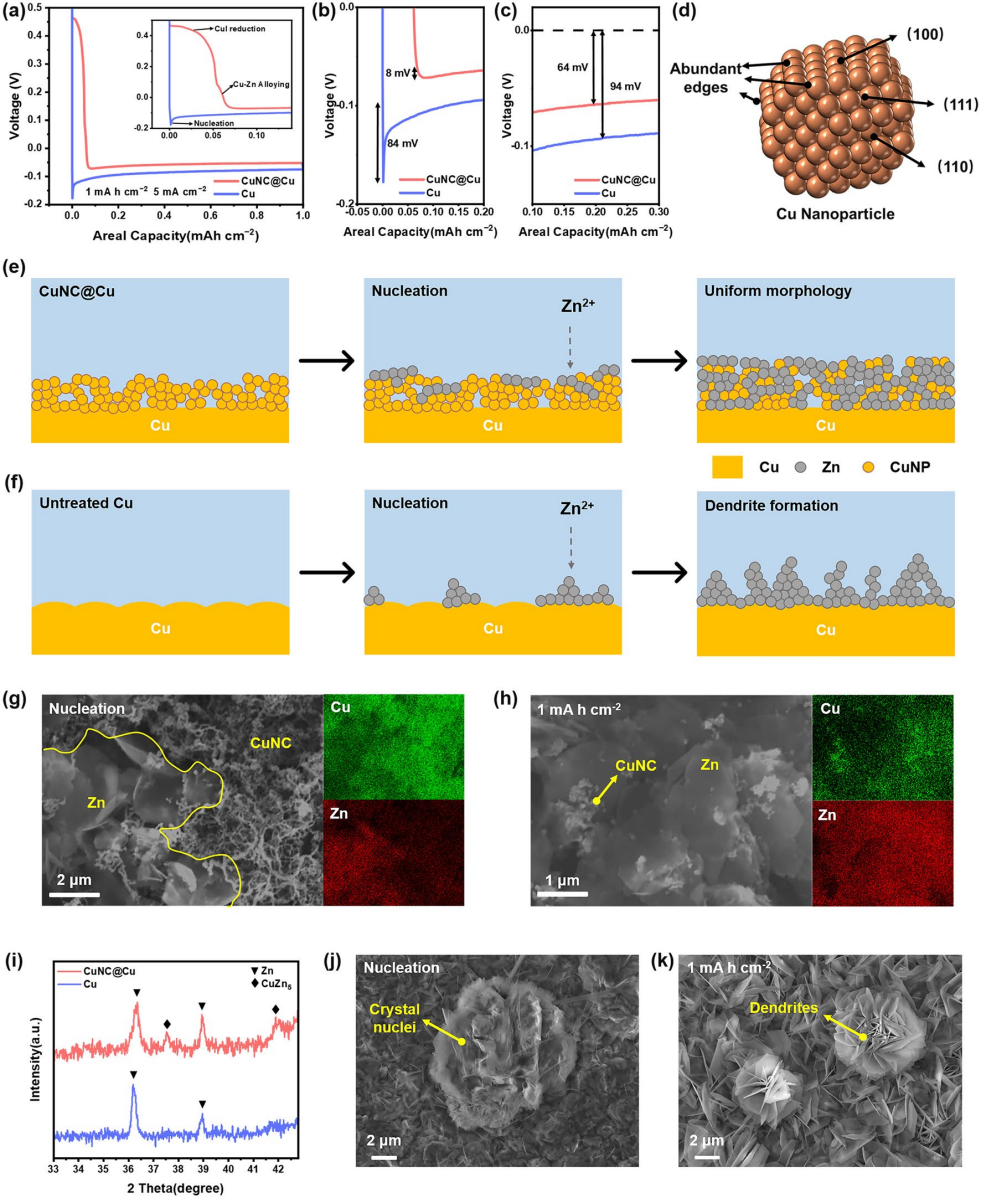
**a** First-cycle galvanostatic deposition curve on CuNC@Cu and Cu electrodes at 5 mA cm^−2^, and the inset shows the different deposition process of these two electrodes. Comparison of **b** nucleation overpotential and **c** deposition potential of CuNC@Cu and Cu electrodes at a current density of 5 mA cm^−2^. **d** Schematic illustration of (100), (110), (111) planes and abundant facet edges of Cu nanoparticles. **e–f** Schematic illustrations of the Zn deposition behavior on CuNC@Cu and Cu electrodes. **g–h** SEM images and corresponding elemental mapping images of Zn deposition morphology on CuNC@Cu electrode at different deposition stages. **i** XRD patterns of CuNC@Cu and Cu electrode after deposition for 12 min. **j–k** SEM images of Zn deposition morphology on Cu electrode at different deposition stages

Next, in situ electrochemical reduction of the iodine-treated copper foil was performed in the Cu/Zn half cell. Notably, the open circuit potential of CuI/Zn half cell is reduced from 0.9 to 0.6 V compared to the untreated Cu/Zn half cell. According to previous reports, CuI, as a conversion electrode material, can undergo a reversible redox reaction with Zn^2+^ to generate Cu [27]. When discharging the cell at a current density of 1 mA cm^−2^, the CuI/Zn half cell exhibits a significant discharge plateau in the voltage range of 0.4–0.5 V (Fig. 3a), which can attribute to the reduction of CuI. In order to demonstrate that the reduction products at − 0.1 V are Cu, the surface layer of I_3_^−^ treated Cu foil was separated from the Cu substrate by transparent adhesive tape. In the XRD pattern (Fig. S2), the I_3_^−^ treated surface Cu foil shows the standard CuI characteristic peaks. After the reduction to 0.1 V, the CuI characteristic peaks are replaced by the characteristic peaks of Cu, indicating the reduction from CuI to Cu. For comparison, no characteristic peaks are observed in the XRD pattern of the transparent adhesive tape from the bare Cu surface. In addition, the cyclic voltammetry (CV) curve (Fig. 2c) of the CuI-Zn half cell further confirms the electrochemical conversion between CuI and Cu [29]. It is worth noting that the redox reaction of Cu to CuI occurs above 0.5 V, thus in order to ensure that Cu is no longer converted to CuI, the cutoff voltage is controlled below 0.5 V in the next half cell tests.

We next employed the *ex-situ* SEM characterization to visualize the evolution of the surface morphology of the Cu foil during iodine treatment (Fig. 2e). As shown in Figs. 2f–h and S3, the iodine-treated Cu foil changed from an intrinsically flat surface to a stack of micron-sized polyhedral particles. After the electrochemical reduction of CuI to 0.1 V, the micron-sized CuI particles were transformed into porous Cu nanoclusters (CuNCs). Actually, the electroreduction of CuI is an outside-in process [29]. Specifically, as shown in Fig. S4, due to the dense structure of CuI, Zn^2+^ can only react with the outer layer of CuI to generate loose Cu nanostructure on the surface, which allows Zn^2+^ to cross the outer layer and further reduce the inner layer of CuI. Thus, with the layer-by-layer reduction of CuI, the CuI micron particles turn into loose Cu nanoclusters. Based on the above results, a nanostructured CuNC@Cu electrode is constructed by a simple in situ electrochemical reduction method.

### Zn Deposition Behavior on CuNC@Cu Electrode

We then performed Zn deposition/dissolution tests and investigated the Zn deposition behavior by *ex-situ* XRD and SEM characterization in the CuNC@Cu/Zn half cells. For the CuNC@Cu electrode, a weak discharge capacity between 0 and 0.1 V is observed in the discharging curve (Fig. 3a). And such a discharge plateau consistently appears in subsequent cycles of CuNC@Cu/Zn half cells (Fig. S5). The corresponding redox peaks between 0 and 0.1 V can also be observed in the CV curve (Fig. 2c), which are attributed to the alloying and de-alloying processes of Cu and Zn [30]. In contrast, the voltage of the untreated Cu/Zn half-cell was instantaneously discharged to 0.01 V, without the alloying plateau. This is because compared to Cu foils, the Cu nanostructures with high specific surface area have more exposed edges of crystal planes in addition to the typical (100), (110), and (111) planes (Fig. 3d), and these edge sites have higher binding energy for Zn atoms [24], thus showing excellent zincophilic properties and lower alloying barrier with Zn [31, 32].

Zn deposition was performed at a current density of 5 mA cm^−2^. For the untreated Cu electrode, a significant nucleation peak appears in the deposition curve with an overpotential as high as 84 mV. In contrast, on the CuNC@Cu electrode, the abundant zincophilic edge sites on CuNC and the formed Cu–Zn alloys can serve as nucleation sites, which greatly reduce the nucleation barriers, resulting in negligible Zn nucleation overpotential (8 mV) in the deposition curve of CuNC@Cu electrode (Fig. 3b). Furthermore, such an ultralow nucleation overpotential and alloying process are also observed in subsequent cycles (Fig. S6), indicating the stability of the CuNC@Cu structure and the reversible formation of Cu–Zn alloys. After nucleation, the Zn deposition morphology was observed by SEM. A large number of crystal nuclei appear on the surface of the untreated Cu electrode (Figs. 3j and S7), with Zn growing undirected around the crystal nuclei and accumulating as large dendrites. In contrast, for the CuNC@Cu electrode (Figs. 3g and S9a), due to a large number of exposed zincophilic sites and ample space for Zn deposition in the CuNC, Zn shows a flaky deposition morphology and horizontally lays on the surface of CuNC, and the corresponding elemental mapping images show the uniform distribution of Zn elements in the CuNC structure, indicating the co-existence of Cu–Zn alloy. The XRD pattern (Fig. S9b) also shows the characteristic peaks of Zn (002 plane at 36.2°) and CuZn_5_ (42.0°), respectively. In addition, it is worthy to note that even in the stable Zn deposition stage, the deposition potential (Fig. 3c) of CuNC@Cu electrode (64 mV) is also significantly lower than that of the untreated Cu electrode (94 mV). And after stable deposition for 30 s, the Zn flakes further grow and uniformly cover the entire surface of CuNC (Fig. S10). After 12 min of deposition, with an area deposition capacity of 1 mAh cm^−2^, the characteristic XRD peaks at 37.8° and 42° of CuZn_5_ are detected on the CuNC@Cu electrode (Figs. 2i and S11d), which further confirm the continued alloying process of CuNC. Meanwhile, the SEM images and corresponding element mapping images (Figs. 2h and S12) show that the Zn flakes are not simply covered on the surface of CuNC, but a large number of Cu nanoparticles are dispersed between the Zn flakes, resulting in a horizontally stacked Zn/Cu composite structure. In contrast, no Cu–Zn alloys are observed in XRD pattern of the untreated Cu electrode (Figs. 2i and S8b). In its SEM images (Figs. 2k and S10a), Zn deposits as vertical blade-like dendrites and further gathers into large flower-like dendrites, which can result in dead Zn and short circuit of the cell.

**Fig. 4:**
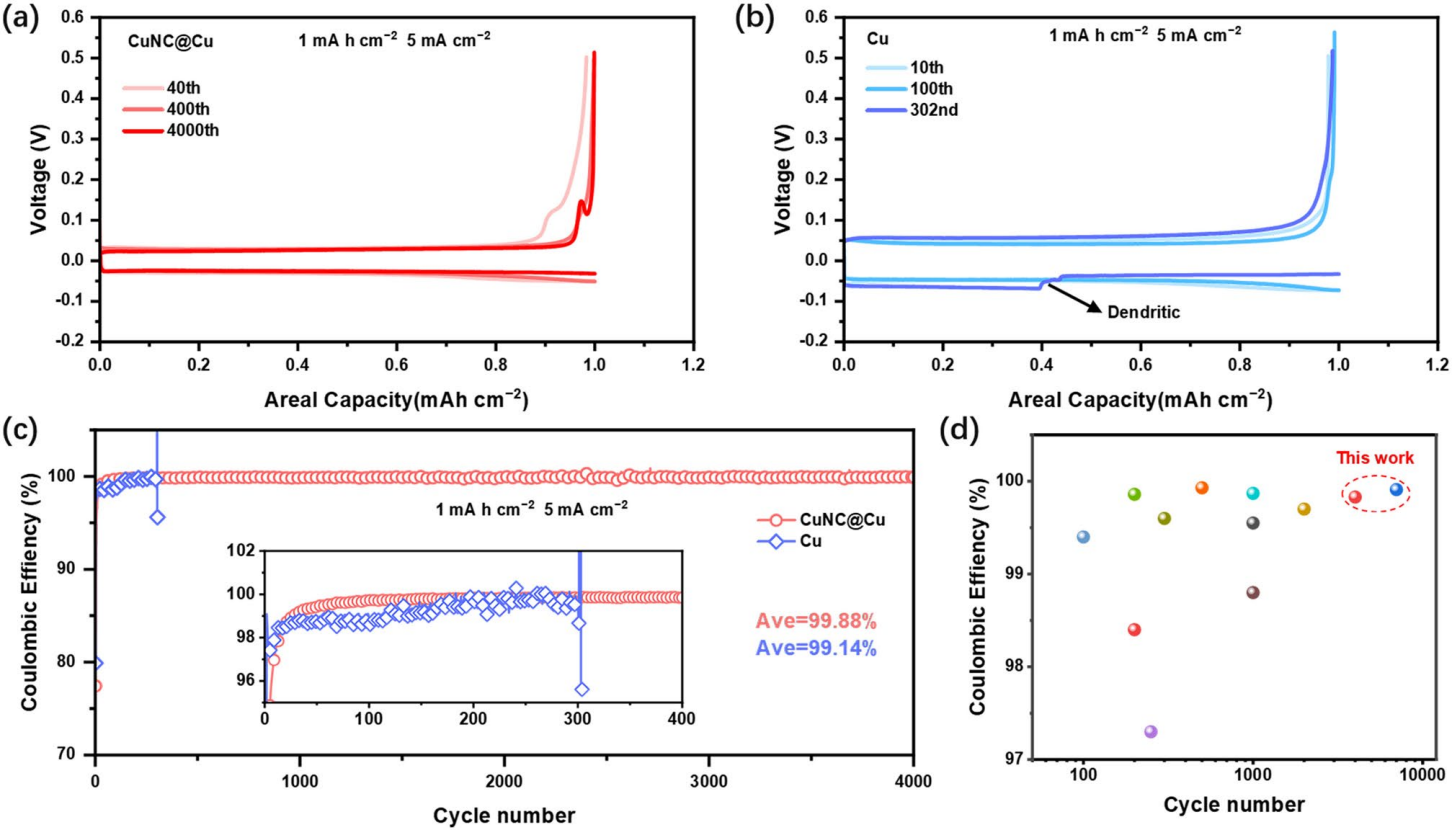
Galvanostatic Zn deposition/dissolution curves of **a** CuNC@Cu and **b** Cu electrodes at selected cycles. **c** CE of Zn deposition/dissolution on CuNC@Cu and Cu electrodes at moderate conditions (1 mAh cm^−2^ and 5 mA cm^−2^). **d** Comparison of ACE and cycle stability of this work with recently reported Zn half cells

The above results confirm that the CuNC@Cu electrode can achieve uniform Zn deposition with ultralow nucleation overpotential. In addition, as an important index to evaluate the reversibility and stability of the anode, we further tested the CE of the half cell. Under moderate conditions (1 mAh cm^−2^, 5 mA cm^−2^), the CuNC@Cu electrode can achieve an ultra-high average CE (ACE) of 99.88% with over 4000 stable cycles (1,700 h, Fig. 4a, c), while the untreated Cu electrode short out at 303^rd^ cycle (Figs. 4b and S13), with an ACE of 99.14%. At higher current densities (1 mAh cm^−2^, 20 mA cm^−2^), the CuNC@Cu electrode delivers even better performance, achieving an ACE of 99.91% over 7,000 steady cycles (800 h, Fig. S14), while the untreated Cu electrode shows 132 stable cycles, with an ACE of 99.42%. The excellent Zn deposition/dissolution reversibility and stability of CuNC@Cu electrode are superior compared with most of the reported studies (Fig. 4d and Table S1) [8, 9, 12, 13, 16, 17, 33–36].

### High-Performance Zn-rich Cathode

Another challenge for anode-free batteries is the lack of high-performance Zn-rich cathodes. For ZnI_2_ cathode, the unique energy storage mechanism based on conversion reaction can provide sufficient Zn^2+^ ions for the anode. However, similar to the iodine cathode [37], ZnI_2_ cathode is suffering from the shuttle effect of iodine species [38], which can cause severe self-discharge and iodine loss, resulting in low CE and dramatic capacity decay[39]. Meanwhile, the poor electronic conductivity of ZnI_2_ greatly limits the utilization of iodine and increases the voltage polarization [40]. To address these issues, we propose a G/PVP heterogeneous structure as a host material for ZnI_2_ cathode.

**Fig. 5:**
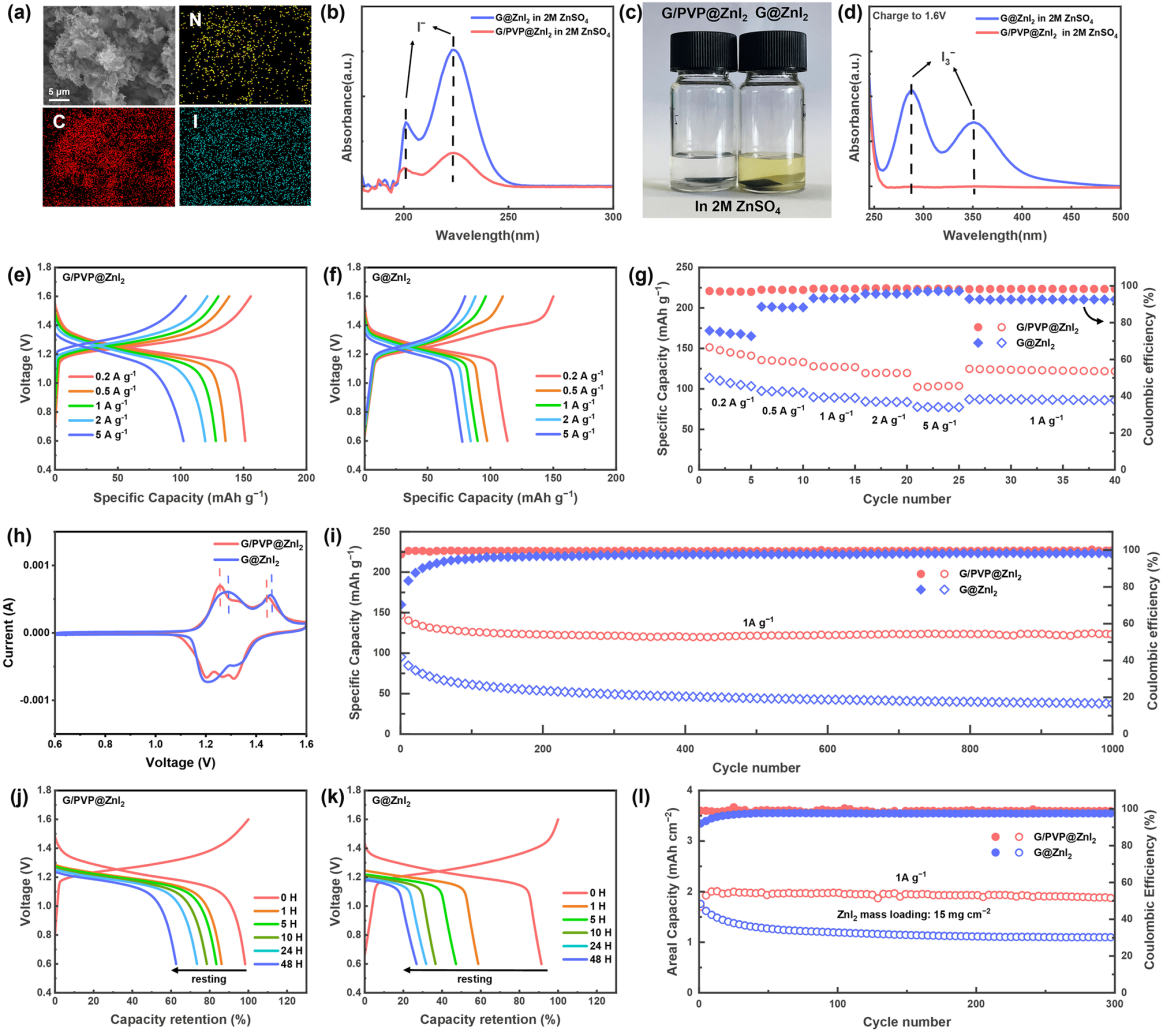
**a** SEM image and corresponding elemental mapping images of G/PVP@ZnI_2_ cathode material. **b** UV–vis absorption spectra of the ZnSO_4_ electrolyte after immersing the G/PVP@ZnI_2_ or G@ZnI_2_ cathode for 24 h (both are diluted by 100 times). **c** Digital photographs of the color change of the ZnSO_4_ electrolyte after immersing the full-charged G/PVP@ZnI_2_/G@ZnI_2_ cathode and separators for 24 h. **d** UV–vis absorption spectra of the ZnSO_4_ electrolyte after immersing the full-charged G/PVP@ZnI_2_ or G@ZnI_2_ cathode for 24 h. **e–g** The rate performance and corresponding GDC curves of the G/PVP@ZnI_2_ and G@ZnI_2_ cathodes. **h** CV curves of the G/PVP@ZnI_2_ and G@ZnI_2_ cathodes at 0.2 mV s^−1^ with the voltage range of 0.6–1.6 V. **i** Long-term cycling test at 1 A g^−1^ of the G/PVP@ZnI_2_ and G@ZnI_2_ cathodes. **j–k** The self-discharge experiments of the G/PVP@ZnI_2_ and G@ZnI_2_ cathodes with a resting time of 0, 1, 5, 10, 24, and 48 h. **l** Cycling stability of the G/PVP@ZnI_2_ and G@ZnI_2_ cathodes at 1A g^−1^ under high areal mass loading

On the one hand, the strong *π*−*π* interaction between G and PVP contributes to the homogeneous and stable structure of G/PVP [41]. PVP can significantly enhance the dispersibility of G in deionized water, therefore, G/PVP exhibits stable and homogeneous dispersion both before and after ultrasonic dispersion, while pure G is heavily aggregated (Fig. S15). Besides, in the SEM images of G/PVP@ZnI_2_ cathode material (Figs. 5a and S16), no flocculent PVP is observed, while the corresponding element mapping image shows the uniform nitrogen distribution of PVP on the graphene surface. This evidence further demonstrates the homogeneous structure of G/PVP [42].Moreover, it is worth to note that, although PVP is water soluble, PVP with an average M_w_ of 30,000 used in this work is difficult to dissolve in the high concentration ZnSO_4_ solution, as shown in Fig. S17a, the PVP solution in 2 M ZnSO_4_ electrolyte is turbid compared to the clarified PVP aqueous solution. Similarly, when the G/PVP electrode was immersed in the 2 M ZnSO_4_ electrolyte for 24 h, as shown in Fig. S17b, the PVP absorption peaks of G/PVP in the UV–vis spectra are negligible compared with the strong absorption peaks of the aqueous solution with the same mass of PVP, indicating that PVP is difficult to dissolve from the G/PVP electrode.

On the other hand, PVP can effectively inhibit the shuttle effect of iodine species, due to the strong electrostatic interaction between PVP and iodide ions. Specifically, PVP chains can form moderate hydrogen bonds with I_n_^−^ (*n* = 1, 3, 5) [43, 44]. For example, after immersing the pre-cycle G/PVP@ZnI_2_ cathode and G@ZnI_2_ cathode in the 2 M ZnSO_4_ electrolyte for 24 h, the UV–vis spectra (Fig. 5b) show that the electrolyte immersed with G/PVP@ZnI_2_ cathode exhibits a significantly lower I^−^ absorption peak. More importantly, the hydrogen bonds get stronger as the number of iodine atoms increases, therefore, the desorption energy barrier of iodine monomers is significantly higher than the adsorption energy barrier [43]. As a result, PVP can effectively inhibit the diffusion of polyiodide to the electrolyte, which is the main cause for the shuttle effect. Disassemble the cells of both cathodes after charging to 1.6 V, yellow iodine species are evident on the separator of the G@ZnI_2_ cathode (Fig. S18), while the separator color of G/PVP@ZnI_2_ cathode is much lighter. After immersing the two samples of cathodes and separators in 2 M ZnSO_4_ electrolyte for 24 h, respectively, as shown in Fig. 5c, the electrolyte immersed with G@ZnI_2_ cathode obviously turns yellow, and the corresponding UV–vis spectra confirm the presence of I_3_^−^ in the electrolyte (Fig. 5d). For comparison, the electrolyte immersed with G/PVP@ZnI_2_ cathode stays colorless and the corresponding UV–vis spectra show negligible I_3_^−^ absorption peak.

Galvanostatic discharging/charging (GDC) tests further demonstrated the suppression of shuttle effect by G/PVP. Figure 5e–g shows the GDC curves and rate performance of G/PVP@ZnI_2_ and G@ZnI_2_ cathodes under various current densities. The CEs of G/PVP@ZnI_2_ cathode is significantly higher than that of the G@ZnI_2_ cathode. At a low current density of 0.2 A g^−1^, G/PVP@ZnI_2_ cathode can deliver an average CE of 96.9%, while the G@ZnI_2_ cathode can only deliver an average CE of 77.2%. Such a low CE results from the severe shuttle effect of the G@ZnI_2_ cathode [45], which is effectively suppressed by the G/PVP@ZnI_2_ cathode. Therefore, the G/PVP@ZnI_2_ cathode exhibits excellent rate performance, delivering an average capacity of 145.6, 137.3, 124.4, 119.6, and 103.1 mAh g^−1^ at 0.2, 0.5, 1, 2, and 5 A g^−1^, respectively, while the G@ZnI_2_ cathode can only deliver an average capacity of 107.8, 96.5, 89.3, 84.0, and 77.5 mAh g^−1^ at 0.2, 0.5, 1, 2, and 5 A g^−1^, respectively. Besides, we also performed self-discharge experiments to demonstrate the suppression of self-discharge behavior by G/PVP. As shown in Fig. 5j–k, after resting for 0, 1, 5, 10, 24, and 48 h, the G/PVP@ZnI_2_ cathode can deliver a capacity retention of 98%, 86%, 83%, 79%, 73%, and 63%, respectively. By contrast, the capacity retention of G@ZnI_2_ cathode is only 91%, 58%, 47%, 37%, 32%, and 27% after resting for 0, 1, 5, 10, 24, and 48 h, respectively. In addition, due to the excellent electronic conductivity and iodine affinity of G/PVP, lower charging voltage and polarization are shown in the charge/discharge curves of G/PVP@ZnI_2_ cathode (Fig. S19). A similar voltage drop of the oxide peak is also observed in the CV curve (Fig. 5h).

In the long cycle test (Fig. 5i), the capacity retention rate of G/PVP@ZnI_2_ cathode can reach 80% after 1,000 cycles, while the G@ZnI_2_ cathode exhibits a sharp capacity decay, with less than 40% capacity retention after 1,000 cycles. In addition, because of the excellent conductivity and stability of G/PVP, we further increased the areal mass loading of cathode. At a high loading of 15 mg cm^−2^, the cathode can deliver an area capacity of 2 mAh cm^−2^ (Fig. S20). Thereafter, no significant capacity decay was observed during 300 stable cycles (Fig. 5l). By contrast, the high mass loading G/PVP@ZnI_2_ cathode shows rapid capacity decay, resulting in significantly lower area capacity. Such an outstanding high areal capacity performance of G/PVP@ZnI_2_ cathode is highly important to increase the energy density of the full cell [46].

### Electrochemical Performance of AFZIB

**Fig. 6:**
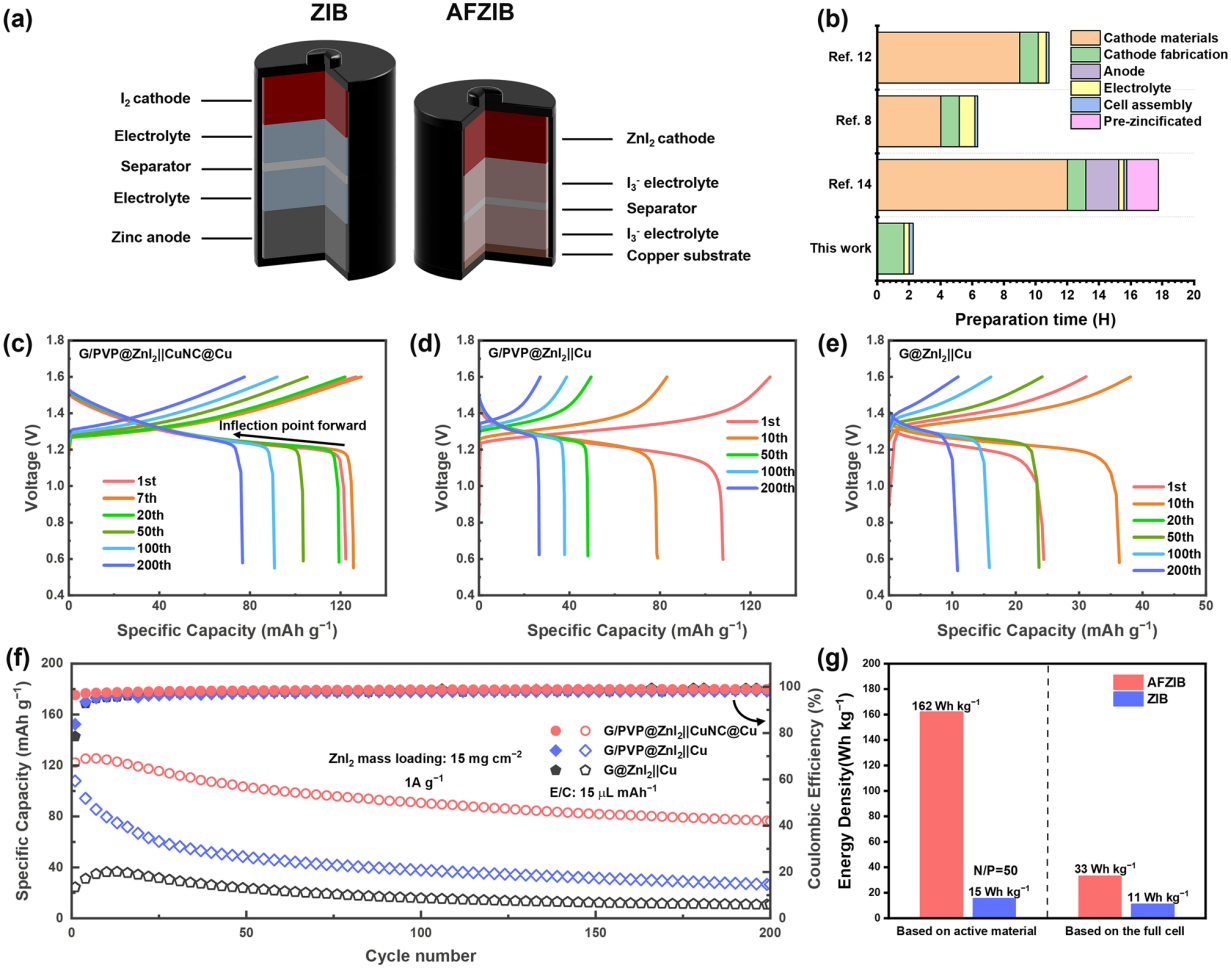
**a** Schematic illustrations of the cell configurations of ZIB and AFZIB. **b** Comparison of preparation time of AFZIB and recently reported AFZBs. **c–e** GDC curves at 1 A g^−1^ of AFZIB with different battery configurations: **c** G/PVP@ZnI_2_ cathode || CuNC@Cu anode, **d** G/PVP@ZnI_2_ cathode || Cu anode, **e** G@ZnI_2_ cathode || Cu anode. **f** Cycling stability of AFZIB at 1 A g^−1^ with different battery configurations. **g** Comparison of gravimetrical energy density of AFZIB and ZIB based on the mass of active material or the mass of full cell

Based on the high-performance ZnI_2_ cathode and the in situ reconstruction method for Cu anode (Fig. 6a), the AFZIB with G/PVP@ZnI_2_ cathode and CuNC@Cu anode can deliver a specific capacity of 125.7 mAh g^−1^ at 1 A g^−1^ (Fig. 6c). It is worth to note that the I_3_^−^ additive in the electrolyte delivers very limited capacity for AFZIB. As shown in Fig. S21, when assembling an AFZIB without ZnI_2_ active substance in the cathode, it only delivers a capacity of about 1.6 mAh g^−1^ (based on the area mass loading of 15 mg cm^−2^, which is the same as the normal AFZIB), which is only 1.3% of the normal AFZIB capacity.

The GDC curve of AFZIB (Fig. 6c–e) is similar to that of ZIB (Fig. 5e). However, as the capacity decays, the voltage inflection point in the discharge plateau becomes more and more forward, indicating that the capacity of AFZIB is mainly limited by the capacity of Zn. Consequently, due to the high reversibility of the CuNC@Cu electrode for Zn deposition/dissolution, AFZIB exhibits excellent cycling stability with a capacity retention of 63.8% after 200 cycles and an initial CE of 96.4% (Fig. 6f). While the capacity retention of AFZIB with G/PVP@ZnI_2_ cathode and Cu anode is only 24.9% after 200 cycles, the capacity decays rapidly at the beginning of the cycle due to the quick loss of Zn, and the initial CE is only 83.8% (Fig. 6d). In the XRD pattern of the surface of Cu foil after cycling (separated from the Cu substrate by transparent adhesive tape, Fig. S22a), significant peak of zinc hydroxide by-product is observed. Besides, if replacing the anode Cu foil with Zn foil after the significant capacity decay of G/PVP@ZnI_2_||Cu battery, the cell capacity returns to the pre-decay level and performs stable cycling (Fig. S22b), which further demonstrates that the main reason for the capacity decay of AFZIB is the loss of Zn. Furthermore, when the cathode material is G@ZnI_2_, due to the shuttle of active material and severe self-discharge, AFZIB can only deliver a capacity of 36.6 mAh g^−1^ and an initial CE of 78.6% (Fig. 6e), with a capacity retention rate of 29.5% for 200 cycles. Compared with recently reported AFZBs with cathode mass loading less than 2 mg cm^−2^, our AFZIB exhibits superior performance even under high mass loading (15 mg cm^−2^) and lean electrolyte (15 μL mAh^−1^) conditions (Table S2). Such a close-to-practice condition is more informative when evaluating the commercialization potential of AFZBs.

More importantly, as the most prominent advantage of AFZIB (Fig. 6f), AFZIB can achieve a gravimetrical energy density of 162 Wh kg^−1^ (based on the total mass of active material), while the gravimetrical energy density of ZIB with 200 μm Zn foil anode is only 15 Wh kg^−1^. Even when the mass of electrode hosts, electrolyte and separator are included in the calculation, the gravimetrical energy density of AFZIB can still reach 33 Wh kg^−1^, which is three times higher than 11 Wh kg^−1^ of ZIB. In addition, commercial electrode materials and simple assembly processes in AFZIB have obvious advantages. The total preparation and assembly process of AFZIB can be limited within 3 h (Fig. 6g), which is much faster than other reported AFZBs, thus reducing the cell production costs and enhancing the practical potential of AFZBs.

## Conclusion

Together, we propose a comprehensive and scalable approach to promoting the electrochemical performance of AFZBs, where low-cost iodine plays an important role in overcoming both the anode and cathode issues. For the anode, the commercial Cu foil surface is in situ reconstructed into zincophilic Cu nanoclusters by I_3_^−^ electrolyte additive. This nanocluster-structure achieves uniform Zn deposition with ultralow nucleation overpotential and high reversibility (average coulombic efficiency > 99.91% over 7,000 cycles). For the cathode, the unique conversion reaction-based energy storage mechanism of iodine enables the ZnI_2_ cathode to provide sufficient Zn^2+^ for AFZB. Then, by optimizing the conductivity and suppressing the shuttle effect through a G/PVP cathode host, a high-capacity, cycling-stable Zn-rich cathode is obtained. Due to iodine enabled high performance of both electrodes, the assembled anode-free Zn iodine full cell delivers stable cycling (capacity retention of 63.8% after 200 cycles) and considerable energy density (162 Wh kg^−1^) under a harsh condition of high cathode mass loading (15 mg cm^−2^) and lean electrolyte addition (15 μL mAh^−1^). More importantly, the low-cost electrode materials and fast-production process demonstrate the scale-up potential of AFZIB, which is crucial for the practical application of AFZBs.

## Supplementary Information

Below is the link to the electronic supplementary material.

Supplementary file1 (PDF 2030 KB)
