# Supplementary material for: Iodine Promoted Ultralow Zn Nucleation Overpotential and Zn-Rich Cathode for Low-Cost, Fast-Production and High-Energy Density Anode-Free Zn-Iodine Batteries
Source: Nanomicro Lett. 2022 Oct 26;14:208. doi: 10.1007/s40820-022-00948-9 (PMC9606174; doi:10.1007/s40820-022-00948-9)
Supplement: Supplementary file 1 — Supplementary file1 (PDF 2030 KB) [file 40820_2022_948_MOESM1_ESM.pdf]

Supporting Information for

Iodine Promoted Ultralow Zn Nucleation Overpotential and Zn-Rich Cathode for Anode-Free Zn-Iodine Batteries

Yixiang Zhang[1], Lequan Wang[1], Qingyun Li[1], Bo Hu[1], Junming Kang[1], Yuhuan Meng[1], Zedong Zhao[1,*] and Hongbin Lu[1,2,*]

[1]State Key Laboratory of Molecular Engineering of Polymers, Department of Macromolecular Science, Fudan University, 2005 Songhu Road, Shanghai 200438, P. R. China

[2]Yiwu Research Institute of Fudan University, Chengbei Road, Yiwu City, Zhejiang 322000, P. R. China

*Corresponding authors. E-mail: 17110440016@fudan.edu.cn (Zedong Zhao); hongbinlu@fudan.edu.cn (Hongbin Lu)

Supplementary Figures and Tables

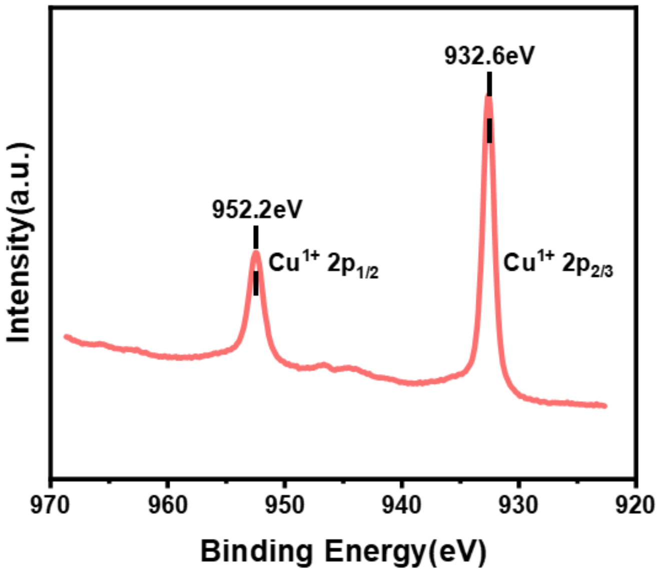

Fig. S1 Cu 2p XPS spectra of iodine-treated Cu foil

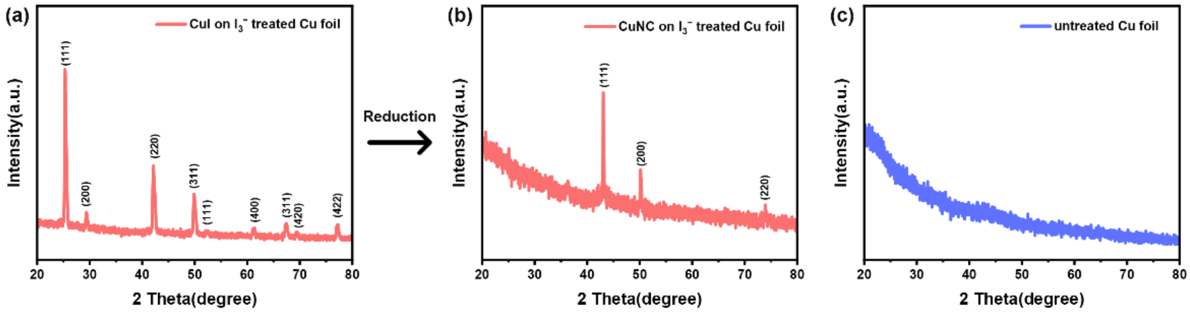

Fig. S2 XRD patterns of the surface layer of **a** CuI@Cu, **b** CuNC@Cu, **c** bare Cu foil, which is separated from the Cu substrate by transparent adhesive tape

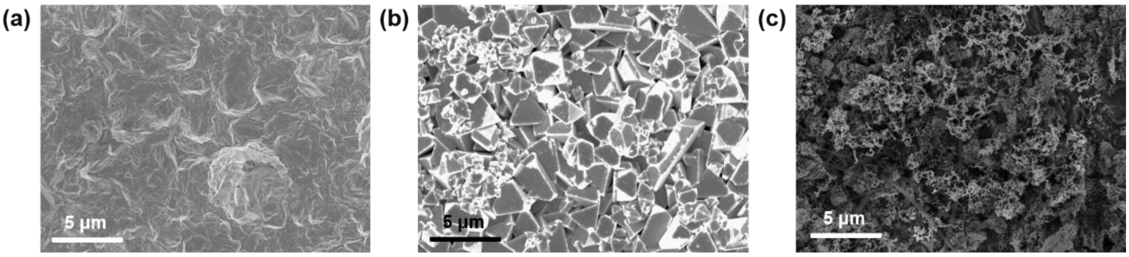

Fig. S3 SEM images of Cu foil **a** before iodine treated, **b** after iodine treated and **c** after reduction to 0.1V

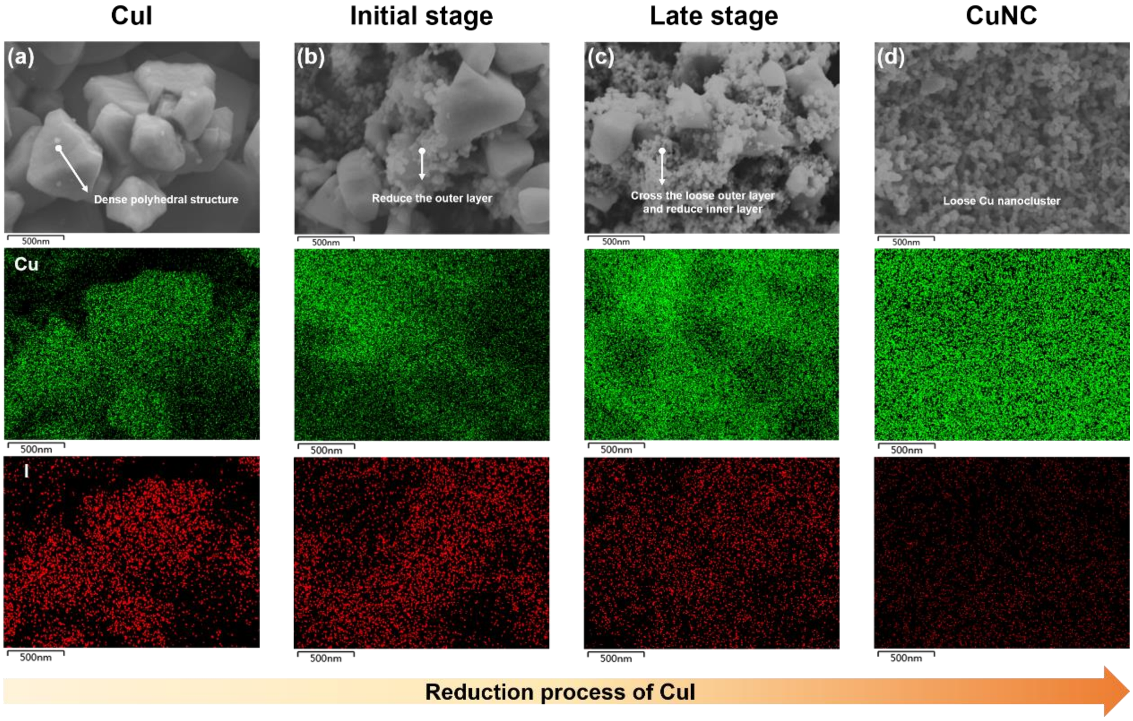

Fig. S4 SEM images and corresponding elemental mapping images of different stages in the CuI reduction process: **a** before reduction, **b** initial stage, **c** late stage and **d** after reduction

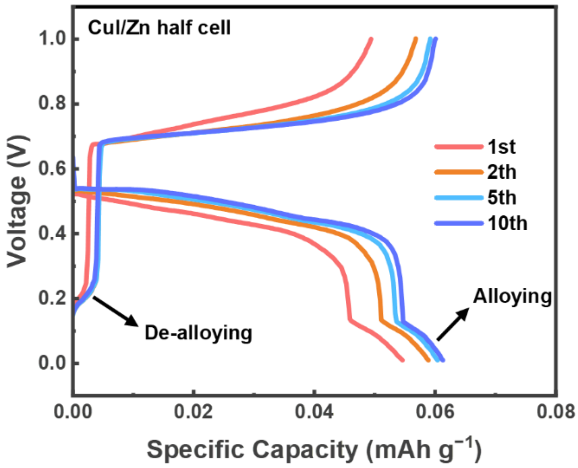

Fig. S5 GDC curves of the CuNC@Cu/Zn half-cell with the voltage range of 0.01-1 V

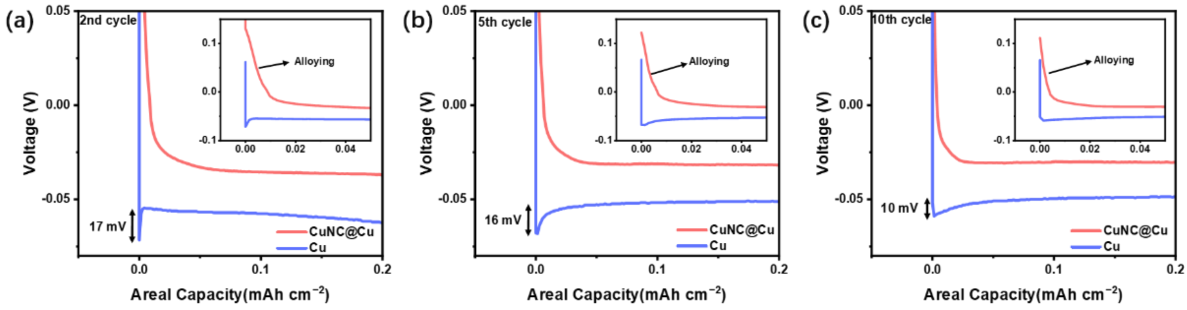

Fig. S6 The nucleation overpotential and alloying process in the **a** 2nd, **b** 5th and **c** 10th cycle galvanostatic deposition curve of CuNC@Cu and Cu electrodes at 5 mA cm^{-2}

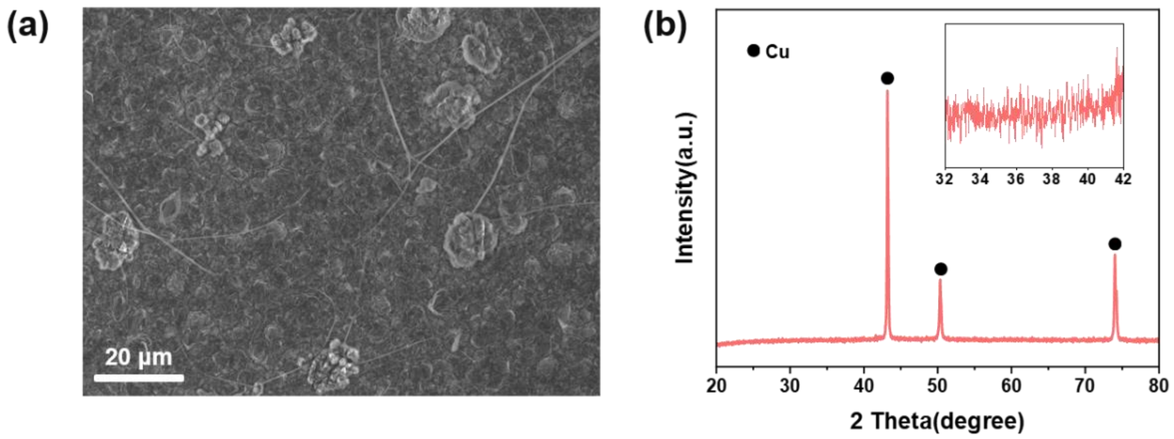

Fig. S7 a SEM images and **b** XRD patterns of Cu electrode after nucleation (Current density: 5 mA cm^{-2})

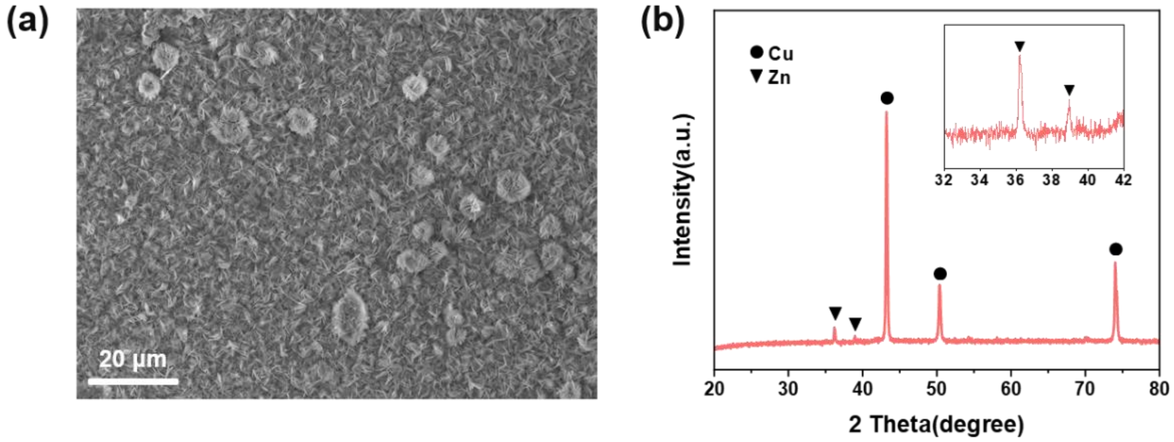

Fig. S8 a SEM images and **b** XRD patterns of Cu electrode after deposition for 12 min (Current density: 5 mA cm^{-2})

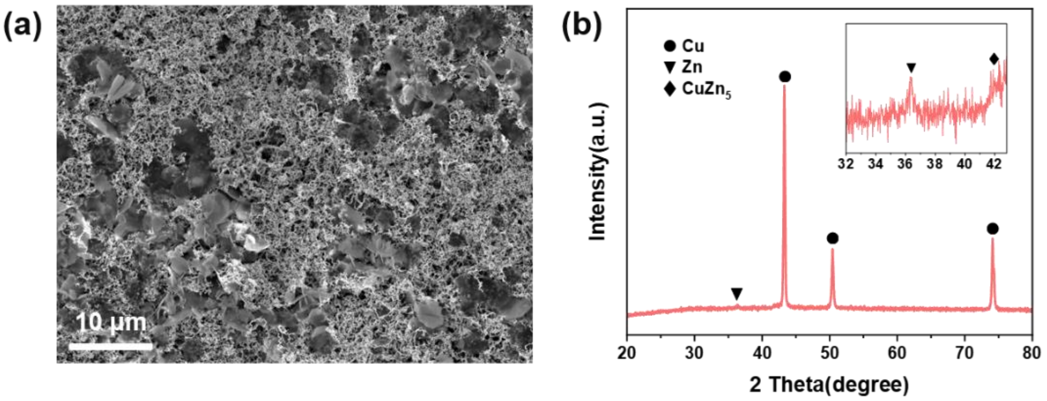

Fig. S9 a SEM images with different magnifications and **b** XRD patterns of CuNC@Cu electrode after nucleation (Current density: 5 mA cm^{-2})

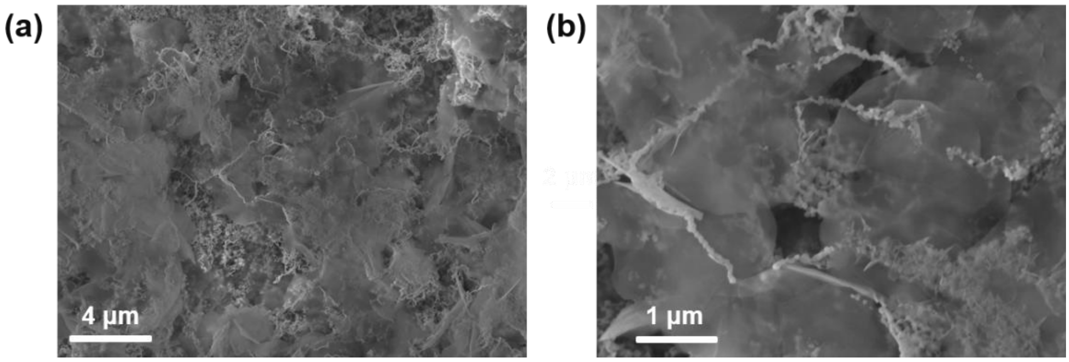

Fig. S10 a-b SEM images with different magnifications of CuNC@Cu electrode after stable deposition for 30s (Current density: 5 mA cm^{-2})

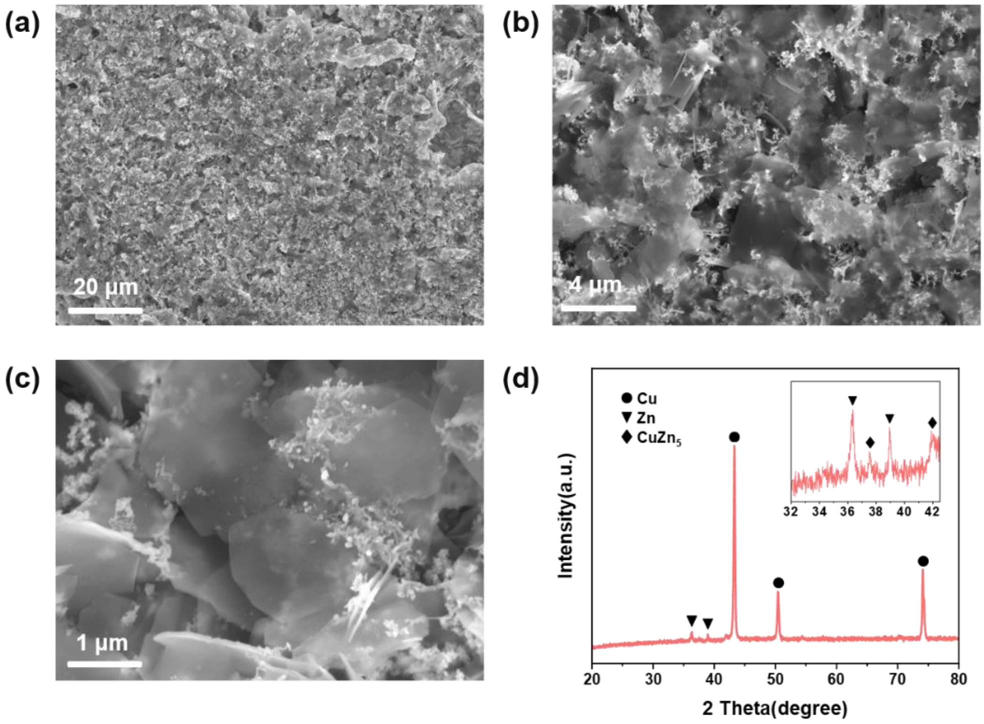

Fig. S11 a-c SEM images with different magnifications and **d** XRD patterns of CuNC@Cu electrode after deposition for 12 min (Current density: 5 mA cm^{-2})

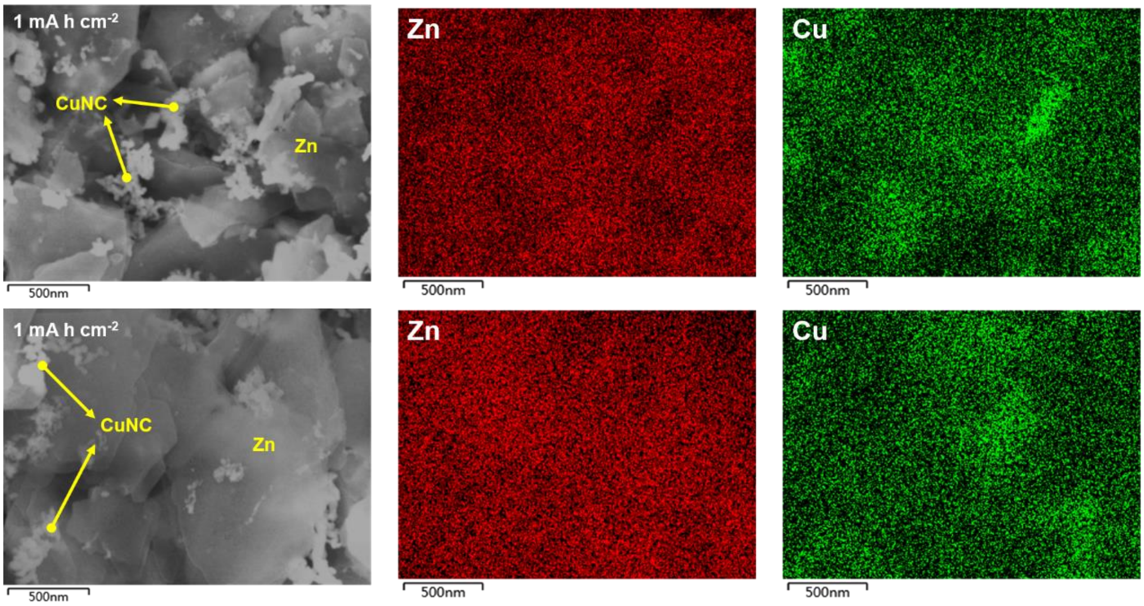

Fig. S12 SEM images and corresponding elemental mapping images of Zn deposition morphology on CuNC@Cu electrode with a Zn deposition capacity of 1 mAh cm^{-2}

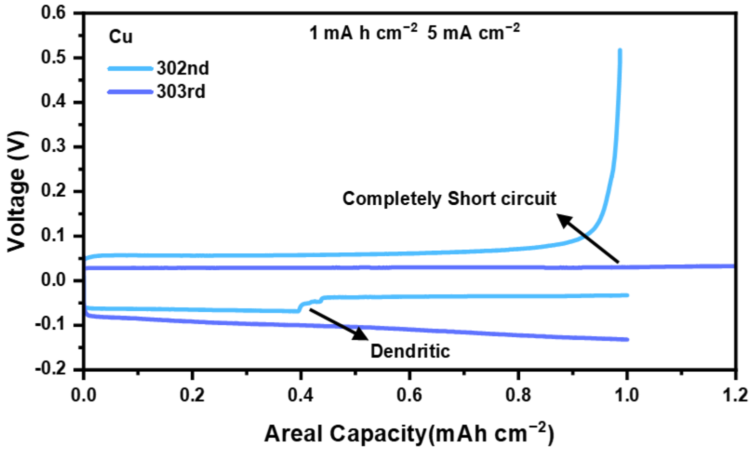

Fig. S13 Galvanostatic Zn deposition/dissolution curves of the 302nd and 303rd cycles of Cu electrodes which is completely short circuit. (1 mAh cm^{-2} and 5 mA cm^{-2})

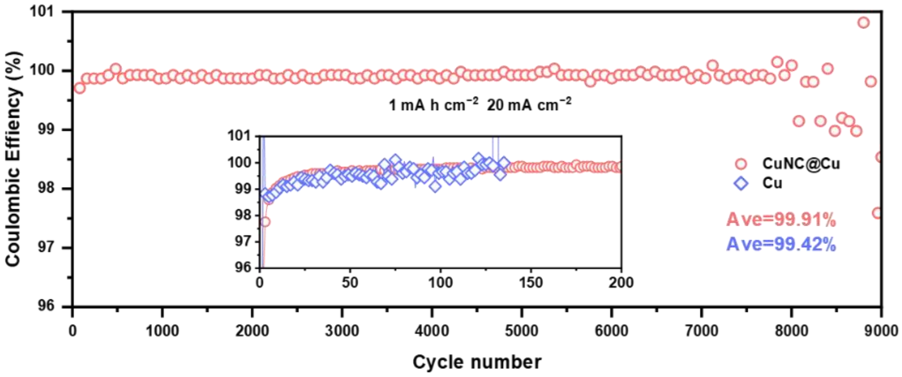

Fig. S14 Coulombic efficiencies of CuNC@Cu and Cu electrodes at high current density. (1 mAh cm^{-2} and 20 mA cm^{-2})

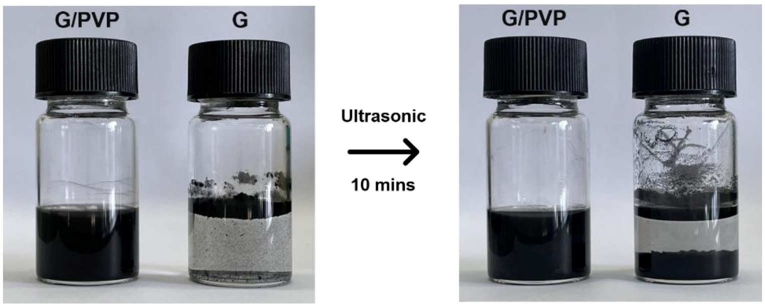

Fig. S15 Aqueous dispersion system of G and G/PVP before and after ultrasonic dispersion for 10 mins

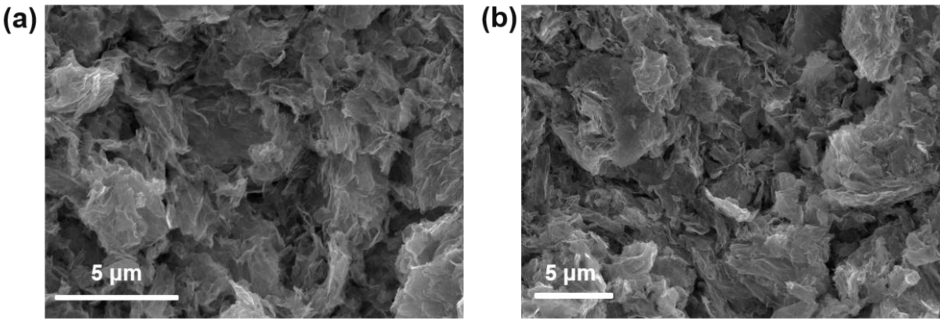

Fig. S16 a-b SEM images of G/PVP@ZnI_2 cathode material

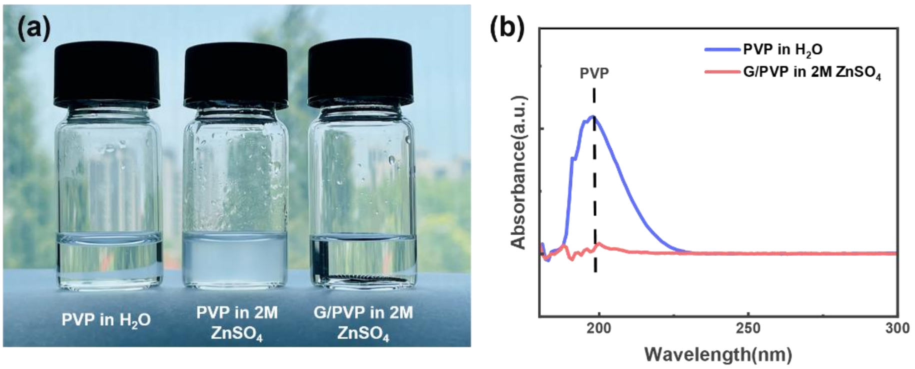

Fig. S17 a Dissolution test of PVP in different systems: PVP in H_2O, PVP in 2 M $ZnSO_4$ and G/PVP electrode in 2 M $ZnSO_4$. **b** UV-vis absorption spectra of the PVP aqueous solution or the $ZnSO_4$ electrolyte after immersing a G/PVP electrode for 24 h (both are diluted by 100 times)

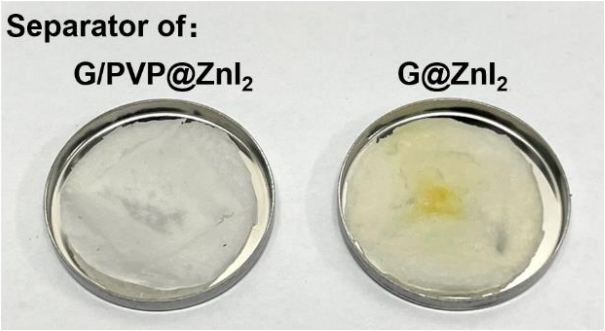

Fig. S18 Separators of the full-charged batteries with G/PVP@ZnI_2 cathode or G@ZnI_2 cathode

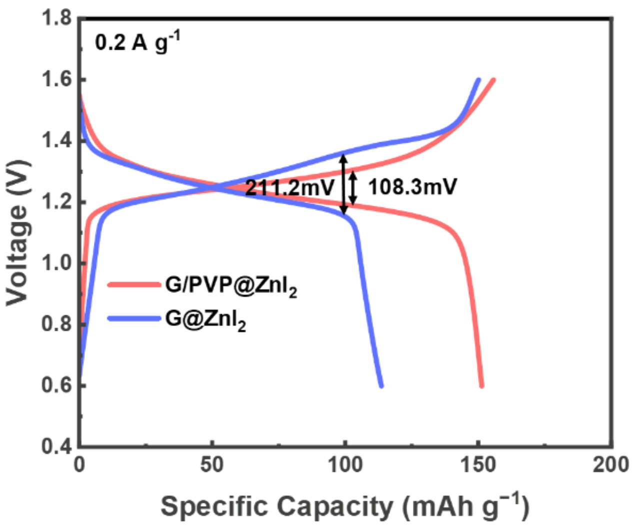

Fig. S19 GDC curves and polarization voltages of the G/PVP@ZnI_2 and G@ZnI_2 cathodes at 0.2 A g^{-1}

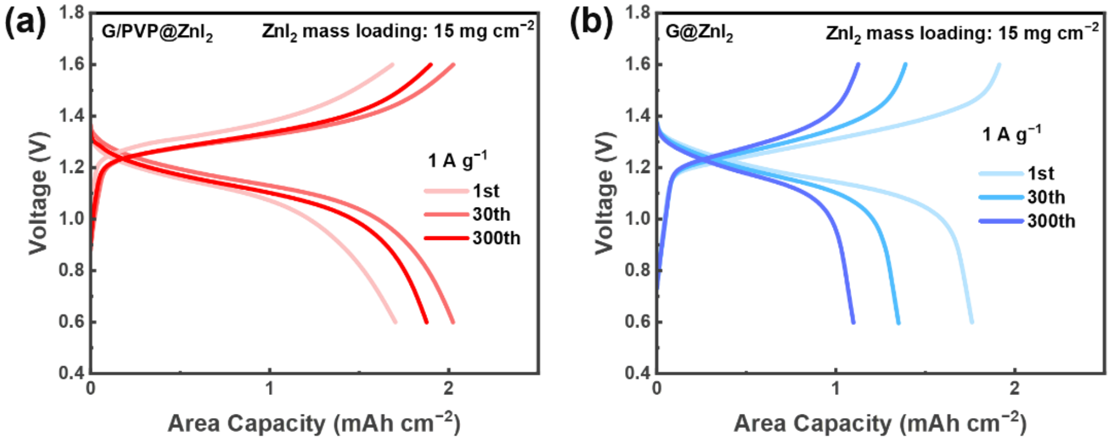

Fig. S20 GDC curves of the **a** G/PVP@ZnI_2 and **b** G@ZnI_2 cathodes at 1 A g^{-1} under high areal mass loading

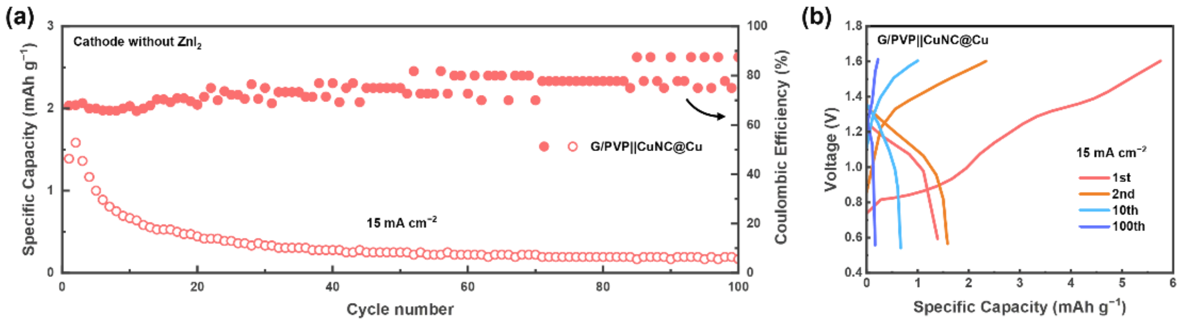

Fig. S21 Cycling curve and GDC curves of AFZIB without ZnI_2 active substance in the cathode. (Current density: 15 mA cm^{-2})

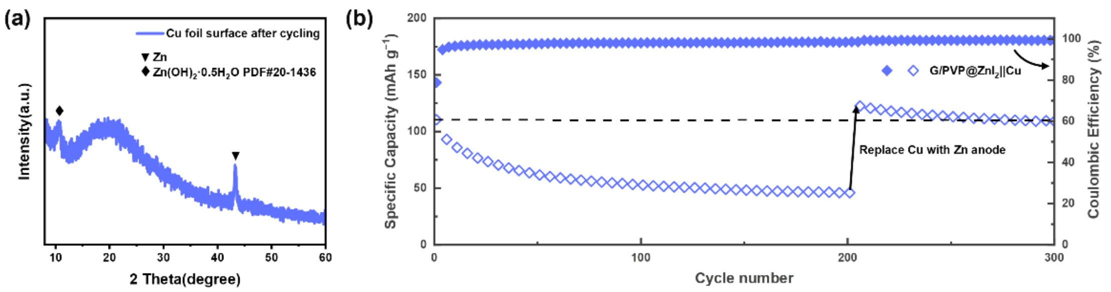

Fig. S22 a XRD pattern of the surface of Cu foil after cycling in AFZIB, which is separated from the Cu substrate by transparent adhesive tape. **b** Cycling curve of AFZIB at 1 A g^{-1} with a battery configuration of G/PVP@ZnI_2 cathode || Cu anode, which has a Cu foil anode replaced by a Zn foil anode after 200 cycles

Table 1 Comparison of ACE and cycle number of this work with recently reported Zn half-cells

Deposited substrate	Electrolyte	Current density (mA cm^{-2})	ACE (%)	Cycle number	Refs.
**CuNC@Cu**	2M $ZnSO_4$+ 5mM ZnI_6	5	99.88	4000	This work
**CuNC@Cu**	2M $ZnSO_4$+ 5mM ZnI_6	20	99.91	7000	This work
**Ti**	2M $ZnSO_4$	40	97.3	250	[S1]
**Ti**	30M $ZnCl_2$ + 5M LiCl	1	99.7	2000	[S2]
**Fe**	2M$ZnSO_4$+0.08M ZnF_2	30	99.87	1000	[S3]
**Cu NBs@NCFs**	2M $ZnSO_4$	5	98.8	1000	[S4]
**C/Cu**	3M $Zn(CF_3SO_3)_2$	1	99.6	300	[S5]
**Cu**	50%PC-sat.	1	99.93	500	[S6]
**Cu**	1M $ZnSO_4$	4	99.4	100	[S7]
**Cu-Ag**	3M $Zn(TFSI)_2$/EMC	0.5	99.86	200	[S8]
**Cu**	2M $ZnSO_4$	2	99.55	1000	[S9]
**ZIF-8-500**	2M $ZnSO_4$	1	98.4	200	[S10]

Table 2 Comparison of capacity and cycling performance of this work with recently reported AFZBs

Electrode (cathode//anode)	Mass loading	Capacity	Cycle number	Decay per cycle	Refs.
**G/PVP@ZnI_2// CuNC@Cu**	15 mg cm^{-2}	125.7 mAh g^{-1} at 1 A g^{-1}.	200	0.19%*	This work
**Prezincated MnO_2//C/Cu**	/	200 mAh g^{-1} at 1 mA cm^{-2}	80	0.40%	[S5]
**$ZnMn_2O_4$//Cu**	1.5-2 mg cm^{-2}	85 mAh g^{-1} at 0.35 A g^{-1}	275	0.07%	[S6]
**$LiMn_2O_4$//Stainless steel**	1.2-1.5 mg cm^{-2}	75 mAh g^{-1} at 0.4 A g^{-1}	100	0.23%	[S3]
**$Zn_3V_3O_8$//Carbon paper**	1.2 mg cm^{-2}	127 mAh g^{-1} at 0.15 A g^{-1}	60	0.68%	[S11]

* under practical applications conditions: high cathode mass-loading: 15 mg cm^{-2} and lean electrolyte addition: 15 μL mAh^{-1}

Supplementary References

[S1] Z. Zhao, R. Wang, C. Peng, W. Chen, T. Wu et al., Horizontally arranged zinc platelet electrodeposits modulated by fluorinated covalent organic framework film for high-rate and durable aqueous zinc ion batteries. Nat. Commun. **12**, 6606 (2021). https://doi.org/10.1038/s41467-021-26947-9

[S2] C. Zhang, W. Shin, L. Zhu, C. Chen, J.C. Neuefeind et al., The electrolyte comprising more robust water and superhalides transforms Zn-metal anode reversibly and dendrite-free. Carbon Energy **3**(2), 339-348 (2020). https://doi.org/10.1002/cey2.70

[S3] Y. An, Y. Tian, K. Zhang, Y. Liu, C. Liu et al., Stable aqueous anode-free zinc batteries enabled by interfacial engineering. Adv. Funct. Mater. **31**(26), 2101886 (2021). https://doi.org/10.1002/adfm.202101886

[S4] Y. Zeng, P.X. Sun, Z. Pei, Q. Jin, X. Zhang et al., Nitrogen-doped carbon fibers embedded with zincophilic Cu nanoboxes for stable Zn-metal anodes. Adv. Mater. **34**(18), 2200342 (2022). https://doi.org/10.1002/adma.202200342

[S5] Y. Zhu, Y. Cui, H.N. Alshareef, An anode-free Zn-MnO_2 battery. Nano Lett. **21**(3), 1446-1453 (2021). https://doi.org/10.1021/acs.nanolett.0c04519

[S6] F. Ming, Y. Zhu, G. Huang, A.H. Emwas, H. Liang et al., Co-solvent electrolyte engineering for stable anode-free zinc metal batteries. J. Am. Chem. Soc. **144**(16), 7160-7170 (2022). https://doi.org/10.1021/jacs.1c12764

[S7] J. Hao, X. Li, S. Zhang, F. Yang, X. Zeng et al., Designing dendrite-free zinc anodes for advanced aqueous zinc batteries. Adv. Funct. Mater. **30**(30), 2001263 (2020). https://doi.org/10.1002/adfm.202001263

[S8] G. Wang, M. Zhu, G. Chen, Z. Qu, B. Kohn et al., An anode-free Zn-graphite battery. Adv. Mater. **34**(29), 2201957 (2022). https://doi.org/10.1002/adma.202201957

[S9] X. Xie, S. Liang, J. Gao, S. Guo, J. Guo et al., Manipulating the ion-transfer kinetics and interface stability for high-performance zinc metal anodes. Energy Environ. Sci. **13**(2), 503-510 (2020). https://doi.org/10.1039/c9ee03545a

[S10] Z. Wang, J. Huang, Z. Guo, X. Dong, Y. Liu et al., A metal-organic framework host for highly reversible dendrite-free zinc metal anodes. Joule **3**(5), 1289-1300 (2019). https://doi.org/10.1016/j.joule.2019.02.012

[S11] J. Wu, Q. Kuang, K. Zhang, J. Feng, C. Huang et al., Spinel $Zn_3V_3O_8$: a high-capacity zinc supplied cathode for aqueous Zn-ion batteries. Energy Storage Mater. **41**, 297-309 (2021). https://doi.org/10.1016/j.ensm.2021.06.006
